# Comparative Transcriptome and Sugar Metabolism Analysis Reveal Regulatory Networks During Bud Dormancy Release in *Prunus mume*

**DOI:** 10.3390/plants15091379

**Published:** 2026-04-30

**Authors:** Wenhui Cheng, Man Zhang, Tangchun Zheng, Jingli Zhang, Qixiang Zhang

**Affiliations:** 1 Beijing Key Laboratory of Ornamental Plants Germplasm Innovation & Molecular Breeding, National Engineering Research Center for Floriculture, Beijing Laboratory of Urban and Rural Ecological Environment, Engineering Research Center of Landscape Environment of Ministry of Education, Key Laboratory of Genetics and Breeding in Forest Trees and Ornamental Plants of Ministry of Education, School of Landscape Architecture, Beijing Forestry University, Beijing 100083, China; cwh13452454084@bjfu.edu.cn (W.C.); zhengtangchun@bjfu.edu.cn (T.Z.); 2 College of Landscape Architecture and Horticulture, Yunnan Agricultural University, Kunming 650201, China; Jl200812@yeah.net

**Keywords:** floral bud dormancy release, *Prunus mume*, sugar metabolism, transcriptome, WGCNA

## Abstract

Sugars play a pivotal regulatory role in floral bud dormancy release in *Prunus mume*, a process that critically determines subsequent flowering time. However, the precise molecular mechanisms linking sugar metabolism to this developmental transition remain poorly understood. To address this gap, we integrated physiological profiling and transcriptomic sequencing using two cultivars with contrasting flowering phenologies: the early-flowering ‘Chaotang Gongfen’ (CTGF) and the late-flowering ‘Shichu Jin’ (SCJ). Exogenous sugar treatments were applied separately to floral buds of the cultivar ‘Yilian’ to evaluate the effect of sugars on dormancy release. During dormancy release, glucose and sucrose contents increased progressively and showed significant positive correlations with bud break rates in both CTGF and SCJ (r > 0.75). Consistently, exogenous application of glucose and sucrose significantly accelerated bud break in ‘Yilian’, whereas mannose exhibited an inhibitory effect. Transcriptome analysis of CTGF and SCJ revealed significant enrichment of starch and sucrose metabolism, hormone signal transduction, and stress-responsive pathways. Key metabolic genes, notably the α-amylase gene *PmAMY1-2* and the cell wall invertase genes *PmCWINV1/4*, were upregulated during this transition. Weighted gene co-expression network analysis (WGCNA) further identified *PmFRK4*, *PmSUS6*, and the aforementioned invertases as candidate genes within a sugar-associated regulatory module. Collectively, these findings support a model in which glucose and sucrose accumulation promotes endodormancy release via the transcriptional activation of starch and sucrose catabolic pathways. This study provides a theoretical framework for deciphering dormancy regulation in woody perennials and offers potential targets for the precise manipulation of flowering time.

## 1. Introduction

*Prunus mume* (mei) is an important ornamental and fruit tree species in early spring, with its timely flowering largely dependent on the successful release of flower bud dormancy [1,2]. This physiological process not only ensures uniform flowering and aesthetic value but also influences fruit production [3]. Sugars are recognized as key regulators of dormancy release, functioning both as energy sources and signaling molecules [4,5]. However, the molecular mechanism by which sugar metabolism regulates this process in mei has yet to be fully understood.

In woody plants, bud dormancy is an adaptive strategy that has evolved to enhance survival under unfavorable environmental conditions [6]. Based on the underlying causes, bud dormancy can be classified into three types: paradormancy, endodormancy, and ecodormancy [7,8]. The release of bud dormancy is regulated by a complex interplay between internal genetic programs and external environmental factors, with low temperature playing a particularly critical role [9,10]. Recent advances have revealed that this transition involves a synergistic network of energy metabolism reprogramming, phytohormone signaling shifts, and redox homeostasis adjustment [4,11]. Within this network, carbohydrate metabolism dynamics serve as a core driver [12]. Studies in tree peony have demonstrated that chilling-induced dormancy release is closely linked to respiratory metabolic pathway shifts, particularly the activation of the pentose phosphate pathway [13]. Furthermore, a recent metabolomics study in tree peony revealed that starch degradation and Embden-Meyerhof-Parnas (EMP) pathway activation are enhanced during dormancy transition, while flavonoids accumulate significantly at the ecodormancy stage, suggesting that primary and secondary metabolism are coordinately remodeled to activate bud break [14]. In peach, this process is tightly coupled with sugar metabolism remodeling, concomitant with a decline in the ABA/GA hormone ratio and regulated oxidative stress [15,16]. Research in apple further indicates that reactive oxygen species (e.g., H_2_O_2_) accumulation dynamics, rather than basal carbohydrate profiles, significantly correlate with flowering time differences among cultivars [17]. These findings collectively highlight both the conserved features and species-specific complexities of dormancy regulation.

In *P. mume*, previous transcriptomic studies have preliminarily outlined a key molecular framework, suggesting that low-temperature signals induce *CBF* transcription factor expression, which subsequently downregulates dormancy-associated *DAM* genes [18]. However, how this transcription factor-centric regulatory network couples with specific changes in sugar metabolic fluxes within the bud, and how sugar metabolites act as signaling molecules to influence the activity of key pathways like *CBF-DAM* to precisely control dormancy release timing, remains systematically unexplored. Specifically, integrated transcriptomic and metabolomic analyses examining the expression patterns of key sugar metabolism-related genes and the association with metabolite accumulation dynamics during dormancy release are still limited.

Recently, multi-omics integration has emerged as an effective approach for elucidating metabolic regulatory networks in plants. Transcriptome and metabolome association analyses have been successfully employed to explore gene function and clarify regulatory pathways in various fruit trees [19], including muskmelon fruit metabolism [20], kiwifruit flavor formation [21], and fig fruit development [22]. For dormancy research, metabolomics has proven valuable in identifying key metabolic shifts, such as the activation of energy pathways and secondary metabolite accumulation during chilling perception in tree peony [14]. In the present study, we employed different *P. mume* cultivars as materials, integrating transcriptomic and targeted sugar metabolomic analyses to systematically elucidate the carbohydrate metabolic characteristics and the transcriptional regulatory mechanisms during flower bud dormancy release. By comparing dormancy release processes among cultivars, clarifying dynamic changes in key carbohydrate components (starch, sucrose, and hexoses), and constructing gene-metabolite correlation networks via WGCNA, we aimed to identify key modules and critical genes highly synergistic with sugar metabolism dynamics. This study represents the first comprehensive investigation into the synergy between transcriptional regulation and metabolic remodeling underlying bud dormancy release in *P. mume*, ultimately aiming to provide a novel theoretical basis for precise flowering period prediction and improved cultivation management.

## 2. Results

### 2.1. Comparative Analysis of Floral Bud Dormancy Release in Prunus mume Cultivars

**Figure 1:**
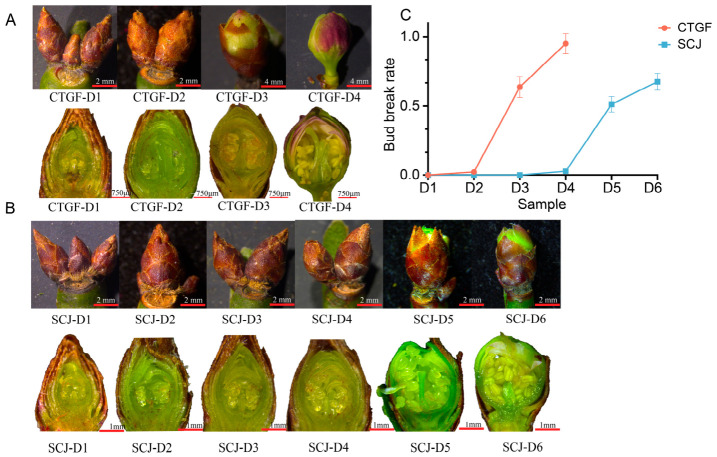
Comparison of dormancy release status in flower buds of different cultivars. (**A**) Flower bud development in the CTGF cultivar from dormancy to dormancy release, (**B**) flower bud development in the SCJ cultivar from dormancy to dormancy release, (**C**) comparison of flower bud break rates between the two cultivars, values represent mean ± SE (n = three biological replicates).

To characterize the dynamics of dormancy release, six sampling time points (14 and 23 December 2022; 3, 12, and 23 January 2023; 3 February 2023) were sequentially designated as D1 through D6. A comparative analysis of bud break rates revealed distinct phenotypic patterns between the two cultivars (Figure 1A,B). The early-flowering cultivar ‘Chaotang Gongfen’ (CTGF) exhibited bud break rates of 0% and 2.32% at D1 and D2, respectively, confirming an endodormant state during these early stages (Figure 1C). In contrast, the late-flowering cultivar ‘Shichu Jin’ (SCJ) remained in endodormancy for a more extended period, showing bud break rates of 0%, 0%, 0%, and 2.85% from D1 to D4 (Figure 1C). Dormancy release occurred rapidly in CTGF, with bud break rates surging to 63.63% by D3 and reaching 95.0% at D4 (Figure 1C), signifying the completion of the release process. For CTGF, developmental stages after D4 corresponded to the flowering period, when dormancy had been fully released; thus, dormancy release stages were defined as D1 through D4, with no samples collected for D5 or D6. By comparison, SCJ did not achieve comparable levels until later stages, with bud break rates of 51.23% and 67.42% at D5 and D6, respectively (Figure 1C). The chilling requirements for the two cultivars were calculated using the Positive Utah Model, yielding 248 CU for CTGF and 486 CU for SCJ (Figure S7), which explains the prolonged endodormancy and delayed bud break observed in SCJ. These results suggest that SCJ maintains endodormancy for a longer duration, undergoes slower floral bud development, and consequently requires a more prolonged period of effective chilling accumulation to initiate bud break, which explains its late-flowering phenotype.

To further characterize the phenotypic divergence in flowering intensity and reproductive success, we monitored the timing and density of bloom under natural field conditions in 2023 (Figure S2). The early-flowering cultivar CTGF reached the 5% bloom stage (defined as the initial phase of flowering when 5% of floral buds on a plant have opened) on January 19, which was 23 days earlier than the late-flowering cultivar SCJ (11 February) (Figure S2). Using the criterion of 50% open flowers to define full bloom, CTGF entered this stage on January 23, exhibiting an extremely dense flowering phenotype with abundant floral display. Conversely, SCJ reached full bloom on February 19 and displayed only moderate flower density. Notably, both cultivars showed a complete absence of fruit development under natural pollination, with no viable fruit set recorded in either genotype throughout the observation period.

### 2.2. Sugar Content Dynamics During Floral Bud Dormancy Release in P. mume

**Figure 2:**
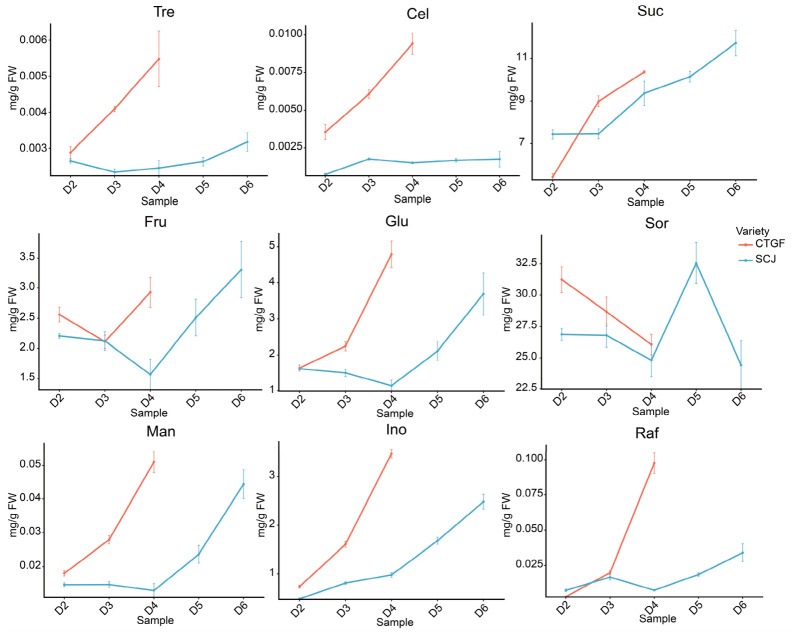
Dynamic changes in sugar content during flower bud dormancy release in different *Prunus mume* cultivars. Sugar contents are expressed on a fresh weight (FW) basis (mg·g^−1^).

To investigate metabolic changes associated with dormancy progression, soluble sugar contents were quantified via GC-MS across the dormancy release period (D2–D6) in both early- and late-flowering *P. mume* cultivars. Distinct temporal accumulation patterns were observed for various sugars, revealing cultivar-specific metabolic dynamics (Figure 2, Table S4).

Trehalose (Tre) content in CTGF exhibited a gradual increase during dormancy release, rising from 0.0028 mg·g^−1^ at D2 to 0.0054 mg·g^−1^ at D4 (Figure 2). In contrast, Tre content in SCJ remained relatively stable from D2 to D4, began to increase at D5 (from 0.00245 mg·g^−1^ to 0.00289 mg·g^−1^), and peaked at 0.00316 mg·g^−1^ at D6 (Figure 2). Cellobiose (Cel) content in CTGF showed a continuous upward trend from D2 to D4, increasing from 0.0035 mg·g^−1^ to 0.0094 mg·g^−1^ (Figure 2). In SCJ, Cel content increased from 0.0007 mg·g^−1^ at D2 to 0.0017 mg·g^−1^ at D3 but remained relatively stable thereafter. Sucrose (Suc), a primary transport and storage sugar, exhibited a steady increase in CTGF from 5.44 mg·g^−1^ to 10.35 mg·g^−1^ over the sampling period (Figure 2). In SCJ, Suc content showed a slower upward trend, rising from 7.43 mg·g^−1^ at D2 to 9.35 mg·g^−1^ at D4, and further increasing to 11.70 mg·g^−1^ by D6 (Figure 2). Fructose (Fru) content followed a similar decreasing-then-increasing pattern in both cultivars. In CTGF, Fru decreased from 2.56 mg·g^−1^ to 2.10 mg·g^−1^, then increased to 2.92 mg·g^−1^ (Figure 2). In SCJ, it decreased from 2.20 mg·g^−1^ to 1.56 mg·g^−1^, then rose to 3.30 mg·g^−1^. Glucose (Glu) content generally increased in both cultivars, with a particularly rapid accumulation observed after endodormancy release (Figure 2). In CTGF, Glu content was 1.62 mg·g^−1^ during endodormancy at D2, increasing to 2.24 mg·g^−1^ at D3 and 4.79 mg·g^−1^ at D4 (Figure 2). In SCJ, Glu content remained relatively stable but slightly decreased from 1.60 mg·g^−1^ at D2 to 1.14 mg·g^−1^ at D4 (Figure 2). It then began to increase at D5 (1.67 mg·g^−1^) and peaked at 3.69 mg·g^−1^ at D6 following dormancy release. D-Sorbitol (Sorbitol) content exhibited divergent trends between the two cultivars (Figure 2). In CTGF, Sorbitol gradually decreased throughout dormancy release (Figure 2). In SCJ, it slightly decreased from D2 to D4, increased to a peak at D5, and then declined. Mannose (Man) and inositol (Ino) contents showed gradual increasing trends during dormancy release in both cultivars, with Man content consistently remaining at approximately one-twentieth of Ino content (Figure 2). Raffinose content followed a pattern similar to that of Tre. In CTGF, raffinose increased markedly from 0.0025 mg·g^−1^ at D2 to 0.097 mg·g^−1^ at D4 (Figure 2). In SCJ, it increased from 0.007 mg·g^−1^ to 0.033 mg·g^−1^ over the same period, with a comparatively smaller magnitude of change (Figure 2).

The remaining sugars (e.g., maltose (Mal), galactose (Gal) and mannose-6-phosphate (Man-6-P)) showed relatively stable levels throughout the sampling period or minor fluctuations without clear temporal trends (Figure S1). The potential roles in dormancy release, remain to be elucidated.

### 2.3. Correlation Analysis of Sugar Content with Floral Bud Break Rate and Among Different Sugars

**Figure 3:**
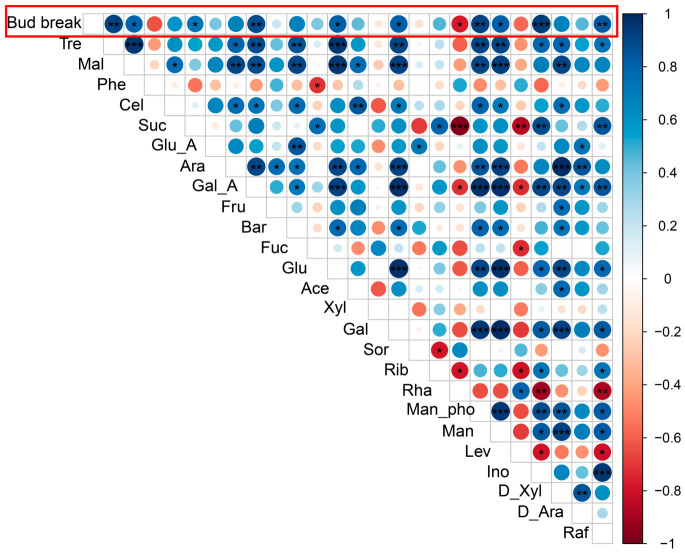
Correlation analysis between flower bud break rate and sugar content. Asterisks indicate statistically significant correlations: * indicates *p* < 0.05, ** indicates *p* < 0.01, *** indicates *p* < 0.001. Red boxes highlight the correlation between flower bud break rate and sugar content.

Correlation analysis revealed that the contents of most sugars were positively correlated with the floral bud break rate. Among these, Tre, Suc, Glu, and Ino exhibited correlation coefficients exceeding 0.75 (Figure 3). Notably, Tre, Manpho, Ino, and galacturonic acid (Gal_A) showed particularly strong positive correlations with the bud break rate, all above 0.9 (Figure 3). In contrast, only rhamnose (Rha) displayed a strong negative correlation, with a coefficient below –0.75 (Figure 3). Most pairwise correlations among different sugars were positive, with the exception of those involving Lev, Rha, and Phe. Specifically, Tre was significantly positively correlated with 11 sugars, including maltose (Mal), Glu, and raffinose (Raf) (Figure 3). Suc showed significant positive correlations with four sugars (e.g., Raf and Ino), but was significantly negatively correlated with Rha and Lev (Figure 3). Meanwhile, Glu exhibited significant positive correlations with 11 sugars, such as Raf, xylose (Xyl), and Ino (Figure 3).

### 2.4. Exogenous Sugar Treatments Validate the Regulatory Roles of Key Sugars in Floral Bud Dormancy Release

**Figure 4:**
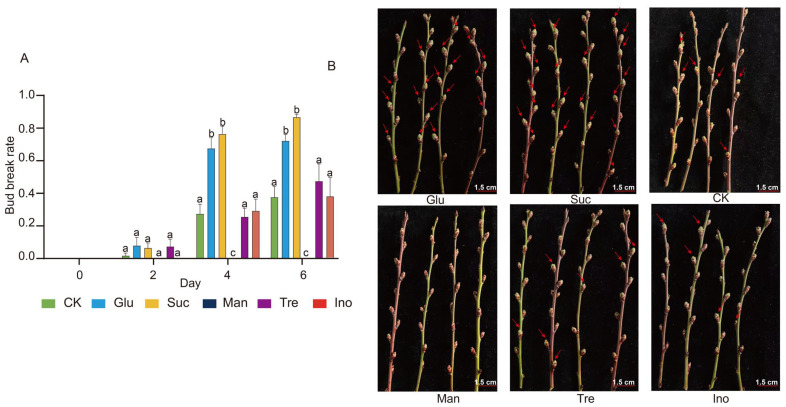
Effects of different sugar treatments on bud break in *P. mume* flower buds. (**A**) Comparison of bud break rates in dormant flower buds of P. mume treated with Tre, Glu, Suc, Ino, Man, and water (CK) after 0, 2, 4, and 6 days, bud break rate (%) = (number of burst buds/total buds per twig) × 100%, values represent mean ± SE (n = three biological replicates, each containing 3 twigs per treatment). Different lowercase letters above the bars indicate statistically significant differences among treatments (one-way ANOVA followed by Tukey’s HSD post hoc test, *p* < 0.05). (**B**) Comparison of flower bud break morphology among different treatments on the 4th day after treatment. The red arrow indicates flower bud break.

To verify whether the sugars identified by correlation analysis—Tre, Glu, Suc, Ino, and Man—directly affect floral bud dormancy release in mei, exogenous spraying treatments were applied to endodormant buds, with water treatment serving as the control. Floral bud break rates were recorded every two days (Figure 4A). During the first two days post-treatment, no significant differences were observed between any sugar treatment and the control (Figure 4A). By day 4, however, distinct treatment effects emerged. Bud break rates under Glu and Suc treatments had reached 67.46% and 76.29%, respectively, both significantly higher than the control (27.35%) (Figure 4A,B). In contrast, Man treatment resulted in a bud break rate of 0%, significantly lower than the control (Figure 4A,B). Tre and Ino treatments yielded bud break rates of 25.28% and 29.20%, respectively, showing no significant difference from the control (Figure 4A,B). The trends observed on day 6 were consistent with those on day 4 (Figure 4A). These results indicate that exogenous application of Glu and Suc significantly promotes flower bud dormancy release, while Man, acting as a potential competitive interaction, suppresses dormancy release.

### 2.5. Comparative Transcriptomic Analysis of the Dormancy Release Process in Two Cultivars

**Figure 5:**
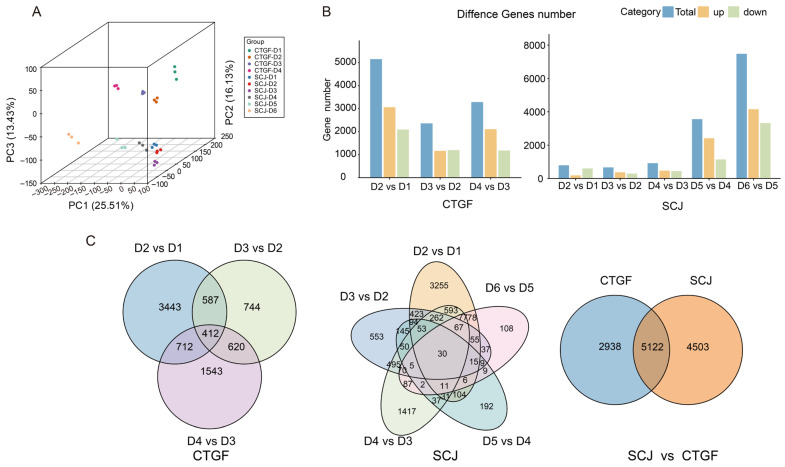
Overview of differentially expressed genes (DEGs) during flower bud dormancy release in *P. mume*. (**A**) Principal component analysis (PCA) of samples, (**B**) number of differentially expressed genes (DEGs) (upregulated and downregulated) between consecutive stages in CTGF (D1–D4) and SCJ (D1–D6), (**C**) venn diagram illustrating the overlap of DEGs between the CTGF and SCJ groups.

To investigate the transcriptional dynamics underlying cultivar-specific differences in dormancy release timing, flower bud samples of the early-flowering cultivar CTGF and the late-flowering cultivar SCJ were collected across endodormancy, ecodormancy, and bud break stages, encompassing one to two key developmental time points per period. Principal component analysis (PCA) of the transcriptomic data revealed clear separation of samples from distinct developmental stages, while biological replicates from the same stage clustered closely together, confirming the reproducibility and stage-specificity of the expression profiles (Figure 5A and Table S6). Using the criteria of |log_2_FC| > 1 and padj < 0.05, a total of 12,563 differentially expressed genes (DEGs) were identified across all comparisons. Comparative analysis of expression dynamics between the two cultivars revealed 5122 common DEGs that were differentially expressed in both CTGF and SCJ during dormancy release (Figure 5C, Table S3).

In CTGF, pairwise comparisons between adjacent developmental stages identified 8060 DEGs (Figure 5C). The transition from D1 to D2, representing the initial stage of dormancy release, exhibited the highest number of DEGs (5153), with a predominance of upregulated genes (3061 upregulated and 2092 downregulated) (Figure 5B). This suggests that the early-flowering cultivar undergoes extensive transcriptional activation at the onset of dormancy release. Throughout the entire process, 412 genes were consistently differentially expressed across three consecutive stages. In contrast, the late-flowering cultivar SCJ displayed a greater total number of DEGs (9625) but with a markedly delayed expression dynamics pattern (Figure 5B). The number of DEGs in the early stages (D2–D4) remained relatively low (ranging from 665 to 919), with large-scale transcriptional activation not occurring until the later stages (D5–D6) (Figure 5B). The D6 vs. D5 comparison showed a peak of 7478 DEGs, indicating a major transcriptional reprogramming event associated with the completion of dormancy release in this cultivar (Figure 5B). A total of 30 genes were consistently differentially expressed across all stages in SCJ (Figure 5C). The 5122 common DEGs identified in both cultivars likely represent a core transcriptional module essential for dormancy release (Figure 5C), whereas the cultivar-specific DEGs may underlie the differential timing of dormancy completion between early- and late-flowering varieties.

### 2.6. GO Functional Classification and KEGG Pathway Enrichment Analysis of DEGs During Floral Bud Dormancy Release in P. mume

**Figure 6:**
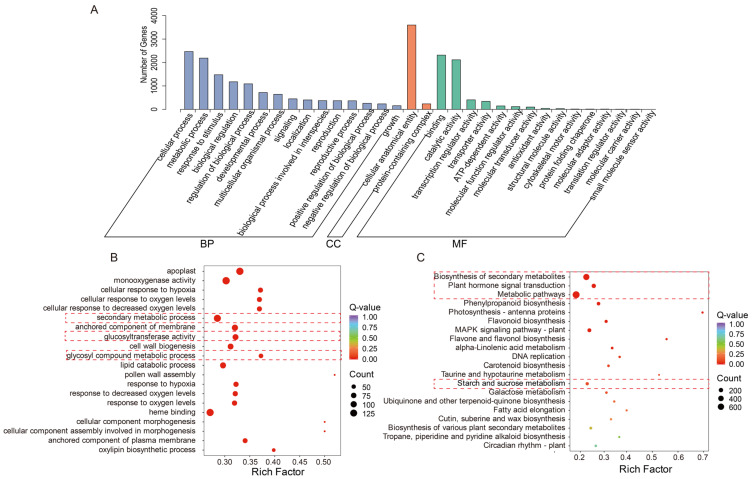
Functional enrichment analysis of DEGs during flower bud dormancy release in *P. mume*. (**A**) GO functional classification of DEGs in CTGF and SCJ, (**B**) GO enrichment dot plot, (**C**) KEGG enrichment dot plot. Red boxes highlight genes involved in key functional categories or metabolic pathways related to sugar metabolism.

Through GO functional annotation and enrichment analysis of the identified differentially expressed genes, this study revealed the active molecular functional landscape during flower bud dormancy release in *P. mume*. These genes were widely distributed across 44 functional branches under the three main ontologies: biological process, cellular component, and molecular function. At the biological process level, differentially expressed genes were significantly enriched in cellular process (2470 genes) and metabolic process (2188 genes) (Figure 6A). Concurrently, a large number of genes were involved in response to stimulus, regulation of biological processes, and biological regulation, suggesting that this process is tightly controlled by endogenous signaling networks and is sensitively responsive to environmental signals (Figure 6A). At the cellular component level, the differentially expressed genes were primarily located in cellular anatomical entity (3600 genes) and protein-containing complex (234 genes), indicating that the cells are undergoing significant structural remodeling, involving organelle functional activation, membrane system reorganization, and functional complex assembly, thereby laying the cellular structural foundation for bud break (Figure 6A). At the molecular function level, gene functions were highly concentrated in binding (2316 genes) and catalytic activity (2116 genes), forming the biochemical basis for metabolism and signal transduction (Figure 6A). The significant enrichment of transcription regulator activity (406 genes), transporter activity (306 genes), and ATP-dependent activity (146 genes) further depicted a complex molecular scenario characterized by energy-driven processes, material transport, and precisely regulated gene expression (Figure 6A).

In-depth GO enrichment analysis further revealed the activation status of key physiological and biochemical pathways: 123 genes were enriched in UDP-glycosyltransferase activity, and 120 genes in hexosyltransferase activity, suggesting that glycosylation modifications are exceptionally active during the dormancy release phase (Figure 6B). The enrichment of 130 genes in secondary active transmembrane transporter activity, combined with the enrichment of the anchored component of the membrane, indicates the crucial role of transmembrane transport and membrane functional regulation (Figure 6B). Furthermore, gene enrichment was particularly pronounced in various abiotic stress response pathways, such as response to cold (116 genes), response to water (108 genes), and response to oxidative stress (102 genes), suggesting that dormancy release is possibly associated with water status perception and oxidative stress adaptation mechanisms (Figure 6B). Simultaneously, secondary metabolism-related functions like phenylpropanoid metabolic process and monooxygenase activity were also significantly enriched (Figure 6B).

Pathway enrichment analysis based on the KEGG database further mapped the differentially expressed genes into a systematic metabolic and signal transduction network, encompassing 137 specific pathways under 5 major categories and 20 subcategories. Among these, metabolic pathways accounted for 106, highlighting the central role of metabolic reprogramming throughout the process. In the overview of global pathways, metabolic pathways, as a master pathway, enriched 776 genes, while biosynthesis of secondary metabolites enriched 560 genes (Figure 6C). The exceptionally high enrichment of these two pathways unequivocally demonstrates that dormancy release in *P. mume* flower buds is a biological process characterized by extremely active overall metabolism and significantly enhanced secondary metabolite synthesis. Within specific branches of secondary metabolism, phenylpropanoid biosynthesis enriched 102 genes, flavonoid biosynthesis enriched 65 genes, and biosynthesis of various plant secondary metabolites enriched 43 genes (Figure 6C). These pathways are not only involved in plant pigmentation and flavor formation but also play critical roles in antioxidant activity, UV protection, and defense responses. The activation may provide protection for subsequent flower opening and participate in signal modulation. Regarding primary material and energy metabolism, differentially expressed genes were extensively involved in carbohydrate metabolism, such as starch and sucrose metabolism enriching 74 genes and galactose metabolism enriching 50 genes (Figure 6C). The activity of these pathways ensures the effective supply of carbon skeletons, energy, and precursor materials. Environmental signal response pathways were also significantly enriched, such as plant hormone signal transduction enriching 194 genes, suggesting that hormone signals might be involved in regulation (Figure 6C). The MAPK signaling pathway—plant enriched 140 genes, and alpha-Linolenic acid metabolism enriched 33 genes, further underscoring the potential roles of signal transduction and lipid metabolism during the dormancy release process (Figure 6C).

### 2.7. Correlation Analysis Between Sugar Content and Gene Expression Modules Based on WGCNA

To investigate the synergistic regulatory relationship between sugar content and gene expression during the dormancy release process in mei flower buds, this study used the measured Glu and Suc contents along with 5122 common differentially expressed genes to construct gene co-expression modules via Weighted Gene Co-expression Network Analysis (WGCNA). This approach systematically elucidated the potential interaction network between sugar dynamics and transcriptional regulation.

**Figure 7:**
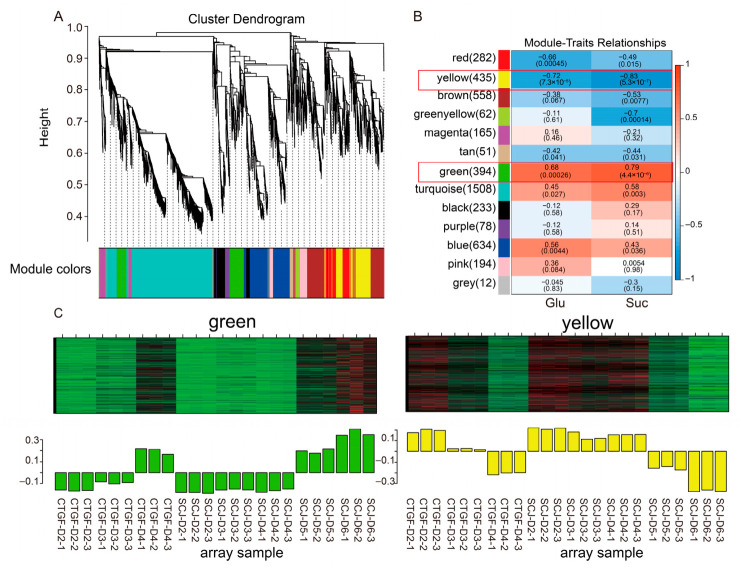
WGCNA of sugar content and differential gene expression. (**A**) Cluster dendrogram of DEG modules, (**B**) correlation analysis between sugar content and gene expression modules. Red boxes highlight gene expression modules significantly associated with the traits, (**C**) green module (positively correlated) and yellow module (negatively correlated) showing significant associations with sugar content.

Sample quality was first assessed through hierarchical clustering. The results showed that biological replicate samples clustered closely according to the developmental stages, indicating good reproducibility, the absence of significant outliers, and conformity with the data consistency requirements for WGCNA. To construct a co-expression network conforming to a scale-free topology, the soft-thresholding power was screened, and a power value of 16 was ultimately selected (Figure 7A). At this value, the network topology fit index reached a high level, and the mean connectivity remained moderate, making it suitable for constructing a biologically meaningful co-expression network (Figure 7A). Based on this parameter, all differentially expressed genes were clustered according to the similarity of the expression patterns across samples. A total of 13 co-expression modules were identified and named with distinct colors. The number of genes varied significantly among modules, with the turquoise module being the largest (containing 1508 genes) and the grey module the smallest (containing 12 genes) (Figure 7B).

Subsequently, the eigengene expression profiles (module eigengenes) of the 13 modules were calculated and subjected to Pearson correlation analysis with the measured Glu and Suc contents to identify gene modules highly coordinated with sugar dynamics (Figure 7B and Table S5). The analysis revealed that multiple modules were significantly correlated with sugar content. Both Glu and Suc contents showed significant positive correlations with the green module (correlation coefficients of 0.68 and 0.79, respectively), the turquoise module (0.45 and 0.58), and the blue module (0.56 and 0.43) (Figure 7B). Conversely, they were significantly negatively correlated with the yellow module (−0.72 and -0.83) and the red module (−0.66 and −0.49) (Figure 7B). Additionally, Suc content exhibited significant negative correlations with the brown module (−0.53) and the green–yellow module (−0.70), whereas Glu showed no significant correlation with these two modules (Figure 7B). Notably, the green module (significantly positive correlation) and the yellow module (significantly negative correlation) were the two modules significantly associated with both Glu and Suc contents. Consequently, key signaling genes will be selected from these two modules for subsequent in-depth analysis (Figure 7C).

### 2.8. Expression Analysis of DEGs Related to Sugar Metabolism

**Figure 8:**
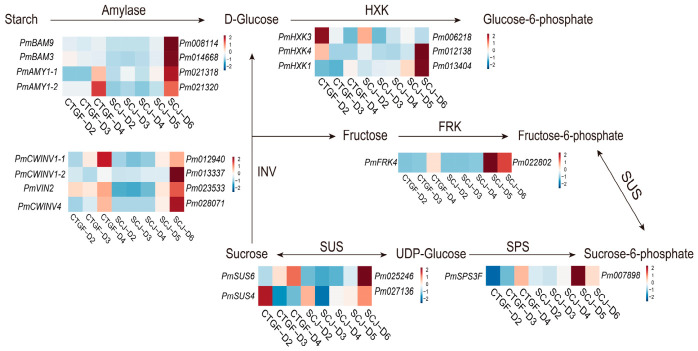
Expression analysis of DEGs associated with sucrose and glucose metabolism.

During the dormancy release process, the contents of glucose and sucrose gradually increased. The expression levels of the starch hydrolase genes *PmAMY1-1* and *PmAMY1-2* peaked at the time of dormancy release in both cultivars (Figure 8). In CTGF, the expression levels of *PmBAM9* and *PmBAM3* did not change significantly throughout the dormancy release process (Figure 8). However, in SCJ, the expression levels of these two genes reached the highest at dormancy release (Figure 8). The expression levels of the invertase genes *PmCWINV1* and *PmCWINV4* gradually increased during dormancy release, consistent with the changing trend of glucose content (Figure 8). In contrast, the expressions of *PmINV2* and *PmINV3* exhibited some fluctuations (Figure 8). Within the sucrose synthase gene family, *PmSUS6* and *PmSUS* showed opposite expression patterns: *PmSUS6* expression gradually increased, potentially promoting sucrose synthesis to facilitate dormancy release (Figure 8). Meanwhile, *PmSUS* maintained a high expression level during endodormancy in CTGF but significantly decreased upon dormancy release (Figure 8). However, in SCJ, *PmSUS* expression showed a trend of initially decreasing and then increasing during dormancy release, suggesting possible regulation by other factors.

Hexokinase is a key enzyme catalyzing the degradation of glucose to glucose-6-phosphate. The expression levels of *PmHXK3* were highest during the endodormancy period in both cultivars and gradually decreased with the accumulation of chilling and the progression of dormancy release (Figure 8). This indicates that maintaining relatively low HXK levels might help inhibit glucose degradation, thereby sustaining sugar homeostasis (Figure 8). Concurrently, the expression levels of enzymes involved in the conversion of active glucose to sucrose-6-phosphate gradually increased (Figure 8), suggesting that the activity of this enzyme might help maintain stable glucose levels through a negative feedback mechanism.

**Figure 9:**
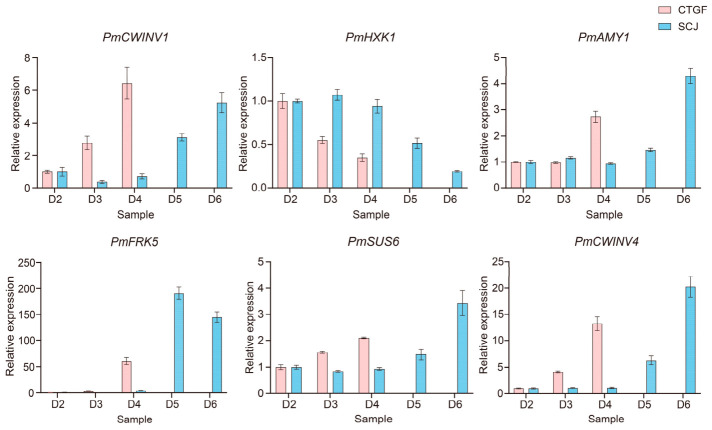
Quantitative real-time PCR analysis of selected genes involved in glucose metabolism.

Based on the co-expression analysis of the green module, key genes related to glucose and sucrose metabolism were identified, including *PmCWINV1*, *PmAMY1*, *PmFRK5*, and *PmSUS6*. Further prediction of the interaction relationships among these genes using the Arabidopsis homologs and the STRING database revealed potential interactions (Figure S6). Among them, *PmSUS6* showed the closest association with other genes, suggesting it may be important for the sugar metabolism regulatory network (Figure S6). The expression patterns of these selected important genes were validated using quantitative real-time PCR (qRT-PCR), and the results were consistent with the transcriptome data (Figure 9).

To explore the association between phytohormone signaling and endodormancy release, we analyzed the expression of ABA-, GA-, IAA-, CTK-, and ET-related genes among the 5122 shared DEGs (Figure S5). In the ABA pathway, biosynthetic genes (*NCED3*, *ABA1*) and the negative regulator *PP2C* remained lowly expressed in both cultivars. *UGT71B6* (ABA glucosyltransferase) declined progressively, with a sharper drop in CTGF, whereas the catabolic gene *CYP707A4* increased, rising faster in CTGF (Figure S5). In the GA pathway, inactivation genes (*GA2OX*, *GA2OX2*, *GA2OX6*) decreased and stayed low, while the biosynthetic gene *GA5* and responsive genes *GASA4* and *GASA6* increased, with higher levels in CTGF than in SCJ (Figure S5). In the IAA pathway, *UGT74B1* (glycosyltransferase), *PIN3* (efflux carrier), and *SAUR76* (early responsive) showed gradual upregulation (Figure S5). In the CTK (Cytokinin) pathway, the degradation gene *CKX3* and the inactivation gene *UGT73C1* declined in both cultivars, more markedly in CTGF. In the ET (Ethylene) pathway, biosynthesis-related genes remained highly expressed, while the transcriptional repressors *ERF-4* and *ERF-9* declined, implying a positive role for ethylene in dormancy release (Figure S5).

## 3. Discussion

**Figure 10:**
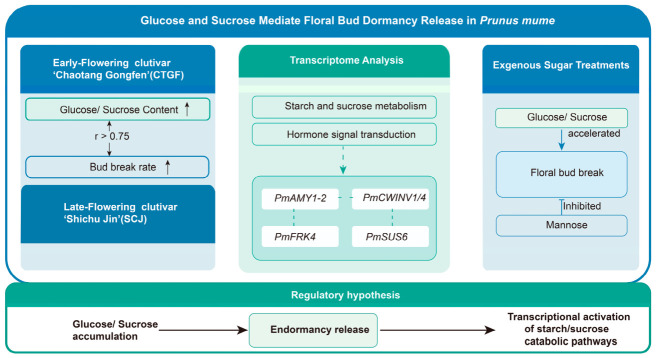
Schematic model depicting the role of sugar signaling in the regulation of floral bud endodormancy release in *P. mume*.

Bud dormancy in woody plants is a highly complex physiological process that ensures survival under adverse conditions and resumption of growth under favorable environmental cues [18,23]. Accumulating evidence indicates that this process is far more than a passive consumption of energy; rather, it is an active and precisely regulated process driven by the reprogramming of carbohydrate metabolism [4,12,19,24]. Using mei as material, this study integrated physiological and biochemical analyses, exogenous treatments, transcriptomics, and weighted gene co-expression network analysis (WGCNA) to reveal the critical role of soluble sugars, particularly glucose (Glu) and sucrose (Suc), as potential metabolic signals promoting dormancy release (Figure 10).

### 3.1. Dynamic Sugar Accumulation Serves as the Metabolic Basis for Mei Floral Bud Dormancy Release

A significant energy demand is a prerequisite for breaking dormancy and initiating bud break. This study found that Glu and Suc contents in mei increased progressively during dormancy release and were highly positively correlated with the bud break rate (Figure 3). Exogenous application of Glu and Suc significantly promoted bud break (Figure 4), consistent with previous findings in poplar [25] and grape [26]. This sugar accumulation primarily reflects the efficient mobilization of carbohydrates, especially starch. In ‘Cuiguan’ pear, starch content showed an inverse relationship with soluble sugar content, indicating that soluble sugar accumulation primarily results from starch degradation [12,24]. Similarly, during low-temperature-induced dormancy release in tree peony, decreased starch and maltose contents were accompanied by increased amylase activity [13,18,27]. In lily bulbs under low-temperature treatment, a significant decrease in starch content was also observed, concurrent with the upregulation of various soluble sugars, including sucrose and trehalose [12]. These cross-species parallel lines of evidence suggest that starch degradation and soluble sugar accumulation are highly conserved metabolic foundations during the dormancy release process in woody plants, providing the necessary energy and carbon skeletons for bud break.

Crucially, the role of sugars has transcended that of mere energy substrates. This study revealed that the accumulation pattern of trehalose (Tre) was highly synergistic with Glu and Suc, showing extremely significant positive correlations with multiple sugar contents (Figure 3). Integrating previous findings, we hypothesize that trehalose, through its precursor trehalose-6-phosphate (Tre6P), may act as a sugar signaling indicator, coordinating carbon metabolism and growth and development by regulating SnRK1 protein kinase activity [28]. In ‘Cuiguan’ pear, the upregulation of the trehalose-6-phosphate synthase (TPS) gene at the Bud break stage also hints at the crucial role of Tre6P signaling [24,29]. Furthermore, sugar signaling extensively cross-talks with plant hormone signaling pathways. In this study, WGCNA identified core modules significantly correlated with sugar content, and KEGG enrichment analysis highlighted pathways such as plant hormone signal transduction and alpha-Linolenic acid metabolism (the jasmonic acid (JA) synthesis pathway) (Figure 6), suggesting potential synergy between sugar signaling and hormones like JA. In tree peony, the upregulation of the key JA signaling pathway transcription factor *PsMYC2* was synchronous with anthocyanin accumulation, and JA has been shown to promote anthocyanin synthesis [30]. These findings collectively depict a complex network where sugar signals act as a important, integrating energy metabolism and hormonal signals (such as GA, ABA, JA) to co-regulate dormancy release.

The more rapid and pronounced upregulation of sugar catabolic genes in the early-flowering cultivar CTGF is consistent with its accelerated endodormancy release, which ultimately manifests as an earlier flowering phenotype. While the upstream genetic factors underlying the differential chilling requirements of these two cultivars remain to be elucidated, our data establish sugar metabolism as a key downstream executor of the dormancy release program.

### 3.2. Transcriptome Profiling Identifies Candidate Regulatory Modules Associated with Floral Bud Dormancy Release

Dormancy release is accompanied by large-scale gene expression reprogramming. In this study, the early-flowering cultivar CTGF underwent massive transcriptional activation in the early stage of dormancy release (D2 vs. D1, 5,153 DEGs), whereas transcriptional activation in the late-flowering cultivar SCJ was significantly delayed, peaking only at the D5-D6 stage (Figure 5). This dynamic difference is closely associated with the varietal differences in chilling requirement and flowering time, revealing at the transcriptomic level that dormancy release is a quantitative accumulation process—gene expression is massively initiated only when chilling accumulation reaches a cultivar-specific threshold. The prevalent enrichment of metabolic pathways and biosynthesis of secondary metabolites indicates that dormancy release is not merely an awakening but a highly active process of synthesis and reconstruction (Figure 6). This is echoed in other studies, such as the significant accumulation of flavonoids (especially anthocyanins and flavonols) in tree peony during the ecodormancy stage [31,32,33], potentially linked to flower coloration and pollen fertility; the enrichment of pathways like phenylpropanoid biosynthesis in ‘Cuiguan’ pear [24] also suggests that secondary metabolites play a role in protecting the bursting buds from environmental stress. Pathways enriched in KEGG, such as carbon metabolism and citrate cycle (TCA cycle), directly point to the core role of energy supply (Figure 6). In ‘Cuiguan’ pear, the dynamic changes in the activities of key enzymes in glycolysis (EMP) and the TCA cycle (GPI, MDH) during dormancy release [24,33,34], and the close association between TCA cycle-related genes and organic acid metabolism in star fruit [19], all corroborate from different angles that the remodeling of carbon and energy metabolism networks is the cornerstone of the dormancy-to-growth transition. Enrichment of terms like cellular anatomical entity and protein-containing complex, along with significant changes in the anchored component of the membrane, implies that the assembly of membrane systems and protein complexes is the structural foundation for bud break (Figure 6). This aligns with the accumulation of UDP-GlcNAc (a cell wall precursor) observed in lily bulbs, which provides raw materials for cell wall construction in nascent buds.

### 3.3. Differential Expression of Key Sugar Metabolism Genes Acts as Molecular Switches Controlling Cultivar-Specific Dormancy Release Progression

The differences in dormancy depth and chilling requirement among cultivars fundamentally lie in the spatiotemporal regulation of genes involved in sugar metabolism. In this study, the distinct dormancy release patterns of the early-flowering cultivar CTGF and the late-flowering cultivar SCJ offered a valuable comparative system for investigating the molecular mechanisms underlying cultivar-specific chilling requirements. Starch hydrolysis is the critical first step. In both cultivars, high expression of amylase genes (*PmAMY*) and cell wall invertase genes (*PmCWINV*) was closely associated with the increase in Glu and Suc (Figure 8), highly consistent with the positive correlation observed between *PpCWINV* expression, chilling accumulation, and budburst rate in pear buds [24]. However, β-amylase genes (*PmBAM9/BAM3*) were highly expressed only during dormancy release in the late-flowering cultivar SCJ (Figure 8), suggesting that certain *BAM* family members may specifically respond to longer dormancy periods or be involved in regulating dormancy depth [18]. The synthesis and decomposition of sucrose must be precisely balanced. Sucrose synthase (SUS) and sucrose phosphate synthase (SPS) are key enzymes controlling sucrose flux. This study revealed opposite expression patterns between *PmSUS6* and *PmSUS* (Figure 8), indicating functional differentiation within the *SUS* gene family. In ‘Cuiguan’ pear, both *SUS* and *SPS* genes exhibited peak activity at the bud break stage, suggesting that sucrose synthesis and breakdown both peak during this period to finely tune sucrose levels [24]. In star fruit studies, high expression of *AcSPS2* and *AcSPS4* during the mature stage correlated with sucrose accumulation trends, further confirming the key role of SPS in sugar accumulation [19]. Hexokinase (HXK) may function as a dual sugar sensor and metabolic gatekeeper [28]. HXK not only phosphorylates hexoses to initiate glycolysis but also acts as a glucose sensor involved in signal transduction [28]. The expression pattern observed in this study—high *PmHXK* expression during endodormancy and low expression during the release phase (Figure 8)—allows us to propose a regulatory model: high HXK activity during endodormancy might consume free Glu and generate the signaling molecule Glu-6-P, thereby maintaining dormancy. As chilling requirements are met, *HXK* expression is downregulated, relieving the capture and signaling inhibition of Glu, leading to Glu accumulation and activation of downstream Bud break programs. This hypothesis finds indirect support in several other species: in peach fruit infected with brown rot fungus, *PpHXK2* expression and HXK activity increased, accelerating sucrose breakdown [35]; heterologous expression of *Prunus HXK3* altered metabolite levels such as G6P and starch in Arabidopsis, enhancing stress tolerance [36]. Collectively, these studies highlight HXK’s key role as a metabolic and signaling hub in plant sugar signaling networks.

Our transcriptome analysis revealed that ethylene biosynthetic genes are actively expressed and that the transcriptional repressors *ERF-4* and *ERF-9* are downregulated during dormancy release, suggesting that ethylene signaling may contribute to the resumption of growth in *P. mume* floral buds. This finding is consistent with long-standing horticultural studies in which calcium carbide is applied to release acetylene, which is subsequently converted to ethylene, to promote flowering in crops such as wheat and cotton [37]. Although the physiological context of temperate woody perennials differs from that of tropical fruit induction, the involvement of ethylene in stimulating metabolic reactivation and cell wall remodeling—potentially via interactions with cell wall invertases (*CWINV*s)—warrants further investigation. Future studies examining the interplay between ethylene signaling, sugar metabolism, and dormancy release in *P. mume* are therefore highly recommended.

By integrating evidence from multiple species, this study reveals the central role of sugar metabolism in regulating flower bud dormancy release in woody plants. Sugars not only serve as sources of energy and carbon skeletons but also act as key signaling molecules through modules like HXK and Tre6P-SnRK1, interacting with plant hormone signaling pathways to collectively initiate and coordinate large-scale gene expression reprogramming, secondary metabolite accumulation, and cellular structural remodeling, ultimately driving dormancy release and the onset of bud break. The potential regulator (e.g., *PmSUS6*) and regulatory modules identified in this study provide an important theoretical foundation and candidate targets for future research dissecting the dormancy regulatory network and for breeding cultivars adapted to different climatic conditions.

## 4. Materials and Methods

### 4.1. Plant Materials and Determination of Floral Bud Dormancy Status

Plant materials were collected from five- to six-year-old mei (*P. mume*) trees cultivated in the field at the Mei Flower Germplasm Resource Nursery of the Meizhou Academy of Agricultural and Forestry Sciences, Meizhou City, Guangdong Province, China (24°16′10.0″ N, 116°4′34.0″ E). Two cultivars with contrasting chilling requirements were selected from the ‘Gongfen’ cultivar group: CTGF (early-flowering) and SCJ (late-flowering). To dynamically monitor the flower bud dormancy release process, samples were collected at six time points from 14 December 2022 to 3 February 2023 (14 and 23 December 2022; 3, 12, and 23 January 2023; and 3 February 2023). All comparisons between cultivars were performed at identical calendar time points. For the early-flowering cultivar CTGF, flowering commenced after January 12, and therefore only four sampling time points were included for this cultivar. At each sampling date, eight to ten one-year-old branches (10–15 cm in length) bearing plump flower buds were randomly collected from each cultivar. The cut ends of the branches were immediately immersed in clean water and transported to the laboratory.

For dormancy status assessment, the bases of the branches were placed in water and maintained in an artificial climate chamber under the following conditions: photosynthetic photon flux density of 200 μmol m^−2^ s^−1^, photoperiod of 12 h light/12 h dark, temperature of 22 ± 1 °C, and relative humidity of 75%. The culture water was refreshed every 2 days, and approximately 1–2 cm was trimmed from the base of each branch to maintain water uptake [38]. After 10 days of cultivation, the number of germinated flower buds on each branch was recorded, and the bud break rate was calculated as: (number of germinated buds/total number of flower buds) × 100%. Following the criteria established by Lang et al. [7,39], a bud break rate of 50% was used as the threshold to determine endodormancy release: samples with a bud break rate < 50% were considered to remain in endodormancy, whereas those with a bud break rate ≥ 50% were considered to have completed dormancy release.

### 4.2. Total RNA Extraction and Quality Assessment

Total RNA was isolated from flower buds using the Omega Plant RNA Kit (Norcross, GA, USA), a reagent specifically designed for plant tissues rich in polysaccharides and polyphenols [40]. The extraction was performed strictly according to the manufacturer’s instructions. The extracted RNA samples underwent rigorous quality control to ensure suitability for transcriptome sequencing. Quantification and preliminary purity assessment were conducted using a Qubit 2.0 Fluorometer (Life Technologies, Carlsbad, CA, USA) [41]. RNA integrity was evaluated by two complementary methods: the RNA Integrity Number (RIN) was determined using an Agilent 2100 Bioanalyzer (Agilent Technologies, Santa Clara, CA, USA), and the integrity of the 28S and 18S ribosomal RNA bands, as well as the absence of genomic DNA contamination, was verified by 1% agarose gel electrophoresis [42]. Only RNA samples that simultaneously met the following criteria were used for subsequent library construction: an A260/A280 ratio between 1.8 and 2.2 (indicating high purity and minimal protein contamination) [41,43], an RIN value ≥ 7.0 [41,43], and gel electrophoresis showing distinct 28S and 18S rRNA bands with clear integrity and no evidence of degradation or DNA contamination [40,42].

### 4.3. Transcriptome Sequencing and Bioinformatics Analysis

Based on the bud break rate statistics and morphological observations, flower buds of CTGF (at 4 key stages) and SCJ (at 6 key stages) during the transition from dormancy to dormancy-release were selected for transcriptome sequencing, with three biological replicates per stage, totaling 30 samples. Paired-end sequencing was performed using the Illumina high-throughput sequencing platform. The obtained raw sequencing data (raw reads) were subjected to quality control using fastp software (v0.23.2) to filter out low-quality sequences and adapters, resulting in high-quality clean reads [44,45]. Using the mei reference genome and its annotation files as a basis [46], the clean reads were aligned to the reference genome with HISAT2 software (v2.2.1) [47]. The featureCounts tool was employed to count the number of reads mapped to each gene [48]. Based on gene length and read counts, TPM (Transcripts Per Million) values were calculated for each gene to normalize expression levels [49]. Subsequent analyses were conducted based on the TPM values and gene count matrix. Differential expression gene (DEG) analysis was performed using the DESeq2 R package (v1.26.0), with screening criteria set as: padj < 0.05 and|log2(Fold change)| > 1 [49,50]. The resulting sets of differentially expressed genes were subjected to gene function annotation and enrichment analysis on the Metware Cloud platform, including KEGG pathway enrichment analysis and GO term enrichment analysis based on hypergeometric distribution tests [51,52,53]. The protein interaction analysis of differentially expressed genes is based on the STRING database and Plant Transcriptional Regulatory Map of known and predicted protein–protein interactions [54]. Use WGCNA for weighted gene co-expression network analysis [55].

### 4.4. Sugar Extraction and GC-MS Analysis

Chemicals and reagents Methanol (MeOH) were purchased from Merck (Darmstadt, Germany). MilliQ water (Millipore, Bradford, Burlington, MA, USA) was used in all experiments. All of the standards were purchased from CNW (Shanghai Anpel, Shanghai, China), IsoReag (Shanghai, China), and TCI (Shanghai, China) [56]. Stock solutions of standards were prepared at a concentration of 2 mg/mL in MeOH. All stock solutions were stored at −20 °C. The stock solutions were diluted with MeOH to working solutions before analysis. Sample preparation and extraction. The freeze-dried materials were crushed using a mixer mill (MM 400, Retsch) with a zirconia bead for 1.5 min at 30 Hz. 20 mg of powder was diluted to 500 μL with methanol: isopropanol: water (3:3:2 *v*/*v*/*v*), vortexed for 3 min, and sonicated for 30 min. The extract was centrifuged at 12,000 rpm under 4 °C for 3 min. 12.5 μL of the supernatant was mixed with 20 μL internal standard (250 μg/mL) and evaporated under a nitrogen gas stream [14]. The evaporated sample was transferred to the lyophilizer for freeze-drying. The residue was used for further derivatization. Derivatization was performed as follows: the sample was mixed with 100 μL of methoxyamine hydrochloride in pyridine (15 mg/mL). The mixture was incubated at 37 °C for 2 h. Then 100 μL of BSTFA was added to the mixture and kept at 37 °C for 30 min after vortex-mixing. The mixture was analyzed by GC-MS (Gas Chromatography-Mass Spectrometry) after diluting to an appropriate concentration [57,58,59,60]. GC-MS analysis Agilent 8890 gas chromatograph coupled to a 5977B mass spectrometer with a DB-5MS column (30 m length × 0.25 mm i.d. × 0.25 μm film thickness, J&W Scientific, Folsom, CA, USA) was employed for GC-MS analysis of sugars. Helium was used as carrier gas at a flow rate of 1 mL/min. Injections were made in the split mode with a split ratio of 5:1 and the injection volume was 1 μL. The oven temperature was held at 160 °C for 1min, and then raised to 200 °C at 6 °C/min, raised to 270 °C at 10 °C/min, raised to 300 °C at 5 °C/min, raised to 320 °C at 20 °C/min and held at the temperature for 5.5 min. All samples were analyzed in selective ion monitoring mode. The ion source and transfer line temperatures were 230 °C and 280 °C, respectively [58,59].

### 4.5. Exogenous Sugar Treatments on Mei Floral Buds

Six to eight dormant branches of *P. mume*, each of uniform length and bearing more than eight flower buds, were selected and sprayed with one of the following solutions: 200 mM Glu, 100 mM Suc, 25 mM Tre, 100 mM Ino, or 100 mM Man [61,62]. Branches treated with distilled water served as the control. After treatment, all branches were placed in a growth chamber under controlled conditions: 24 °C with 12 h of light, followed by 22 °C in darkness for 12 h. Flower bud break rates were monitored and recorded every two days. Experiments were performed with three biological replicates. Data were expressed as mean ± standard error (SE). Statistical analyses were performed using R software (v4.1.0). When one-way analysis of variance (ANOVA) indicated significant differences among groups, post hoc pairwise comparisons were conducted using Tukey’s Honest Significant Difference (HSD) test with the TukeyHSD() function from the built-in stats package [63]. A *p*-value < 0.05 was considered statistically significant. Correlation analyses were conducted using Spearman’s rank correlation coefficient (r) to evaluate the relationships between sugar contents and budbreak percentages. Calculations were performed using the rcorr() function from the Hmisc package in R. Asterisks indicate statistically significant correlations: * indicates *p* < 0.05, ** indicates *p* < 0.01, *** indicates *p* < 0.001.

### 4.6. Quantitative Real-Time PCR (qRT-PCR) Analysis

Quantitative real-time PCR (qRT-PCR) was carried out on the PikoReal platform (Thermo Fisher Scientific, Dreieich, Germany) with three biological replicates to determine the expression levels of six genes involved in sugar metabolism. The thermal cycling conditions were set as follows: initial denaturation at 95 °C for 30 s, followed by 40 cycles of 95 °C for 5 s and 60 °C for 30 s, and a final extension at 72 °C for 30 s. Relative expression levels were calculated using the 2^−ΔΔCt^ method, with protein phosphatase 2A (*PP2A*) as the internal reference gene [63]. The primer sequences used are listed in Tables S1 and S2.

## 5. Conclusions

This study elucidates the physiological and molecular mechanisms underlying differential dormancy release in early- and late-flowering *Prunus mume* cultivars. Phenotypic analysis confirmed that the late-flowering cultivar SCJ exhibits a prolonged endodormancy period and slower bud development compared to the early-flowering CTGF. Sugar profiling revealed that glucose and sucrose accumulate in correlation with dormancy progression, and exogenous application experiments functionally validated these sugars as positive regulators that significantly promote bud break, while mannose suppressed it. Transcriptomic analysis demonstrated that CTGF undergoes immediate and extensive transcriptional activation at dormancy release onset, whereas SCJ displays a markedly delayed expression response. Through WGCNA, we identified specific gene co-expression modules highly correlated with sugar dynamics and pinpointed *PmSUS6* as a candidate gene within the sugar metabolism regulatory network. These findings suggest that floral bud dormancy release in *P. mume* is accompanied by coordinated changes in sugar signaling and transcriptional reprogramming. The delayed transcriptional activation and slower metabolic shifts in SCJ likely contribute to its late-flowering phenotype, providing valuable insights for breeding programs targeting flowering time optimization. Furthermore, the contrasting dormancy behaviors observed between these two cultivars illustrate how intraspecific variability in sugar metabolism and gene regulatory networks can shape phenological traits, thereby offering a framework for assessing the evolutionary adaptation of plants to local climatic conditions.

## Data Availability

The datasets used in this study are publicly available (BioProject: PRJNA1437719). All analyzed data can be found in the article or in the Supplementary Material.

## Supplementary Materials

The following supporting information can be downloaded at: https://www.mdpi.com/article/10.3390/plants15091379/s1, Figure S1. Dynamic changes in the contents of 16 sugars during flower bud dormancy release in different *P. mume* cultivars; Figure S2. Comparison of flowering intensity and bloom stage between early-flowering CTGF and late-flowering SCJ; Figure S3. Comparing flower bud break morphology between treatments at 0 and 6 days after treatment; Figure S4. Determination of WGCNA parameters and quality control of sample data; Figure S5. Expression heatmap of DEGs associated with phytohormone signaling during floral bud endodormancy release; Figure S6. Prediction of protein interaction networks in sugar metabolism and the upstream transcription factor binding networks within the green module in *P. mume*; Figure S7. Chilling accumulation of two cultivars calculated using the Positive Utah Mode. Table S1. List of primers used for quantitative real-time PCR (qRT-PCR) analysis; Table S2. Full-length CDS sequences of genes related to carbohydrate metabolism; Table S3. Comparative analysis of gene expression profiles in two *P. mume* cultivars during dormancy release; Table S4. Trend Analysis of 25 Sugar Compounds during Dormancy Release in *P. mume*; Table S5. Differentially expressed genes assigned to the green and yellow modules in WGCNA; Table S6. Summary of transcriptome sequencing data and quality assessment; Table S7. Correlation analysis between flower bud break rate and sugar content.
