# Supplementary figures and images for: Comparative Transcriptome and Sugar Metabolism Analysis Reveal Regulatory Networks During Bud Dormancy Release in Prunus mume
Source: Plants (Basel). 2026 Apr 30;15(9):1379. doi: 10.3390/plants15091379 (PMC13164620; doi:10.3390/plants15091379)

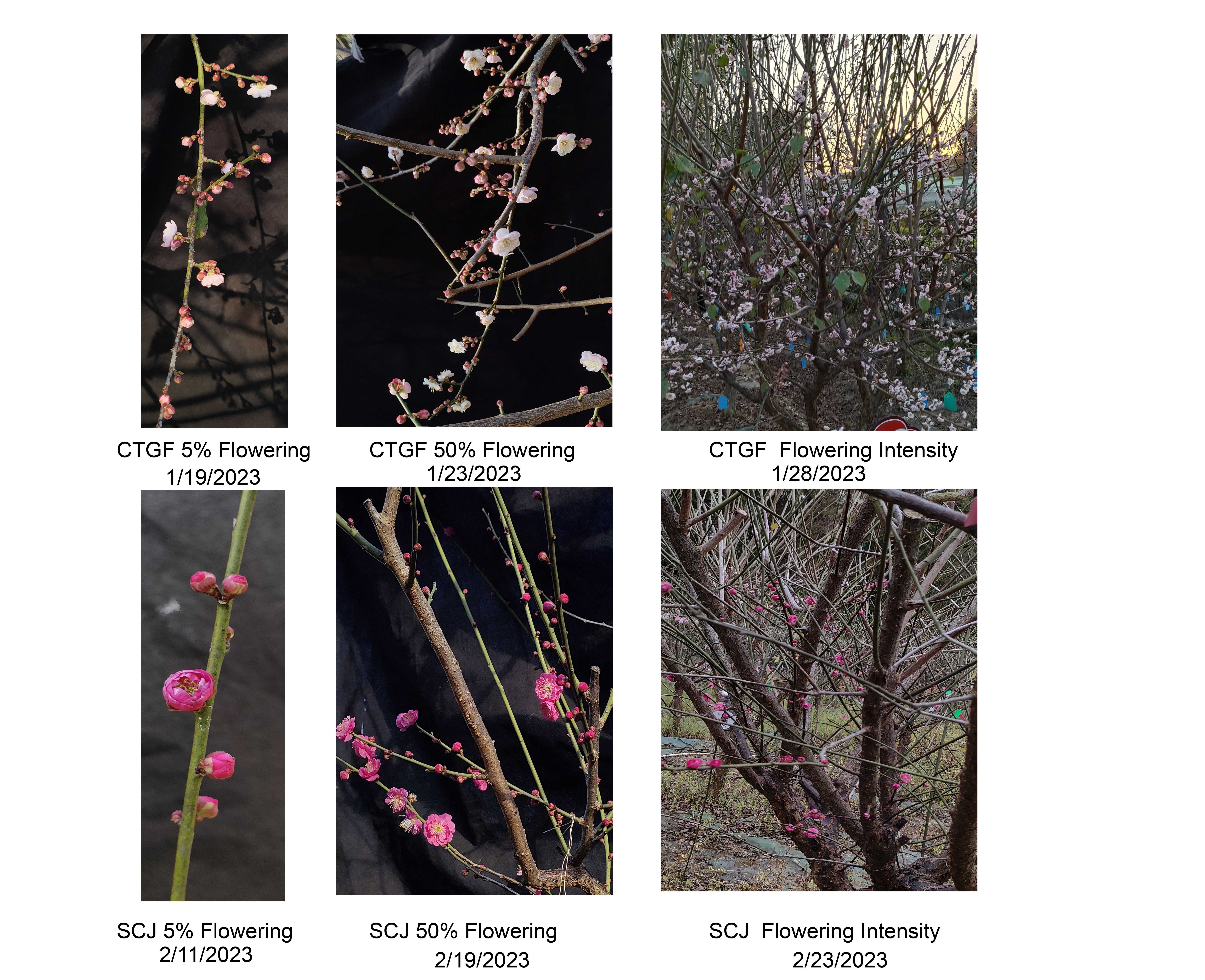

Supplement: Supplementary file 1 [file plants-15-01379-s001.zip › Figure S2.tif]

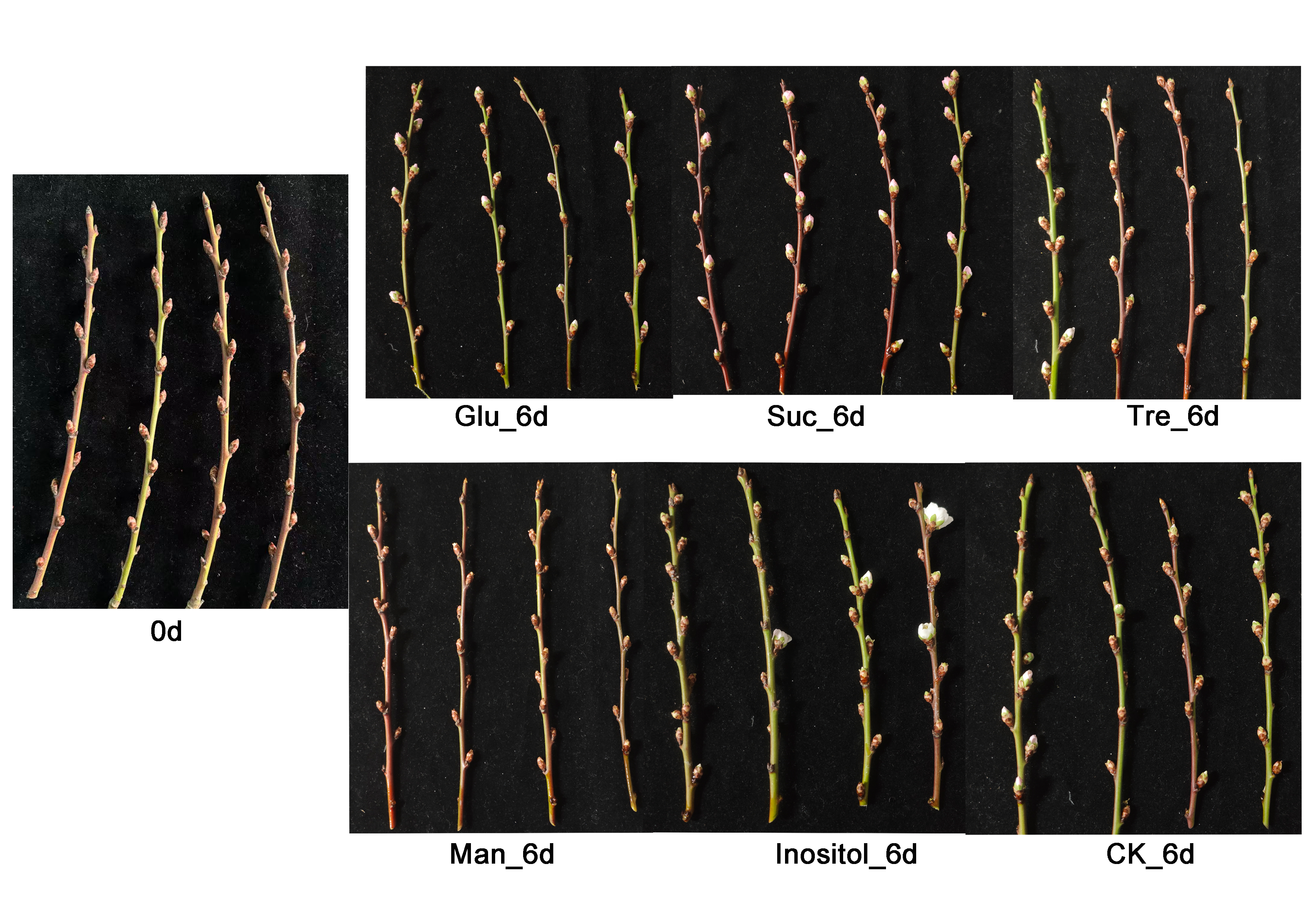

Supplement: Supplementary file 1 [file plants-15-01379-s001.zip › Figure S3.tif]

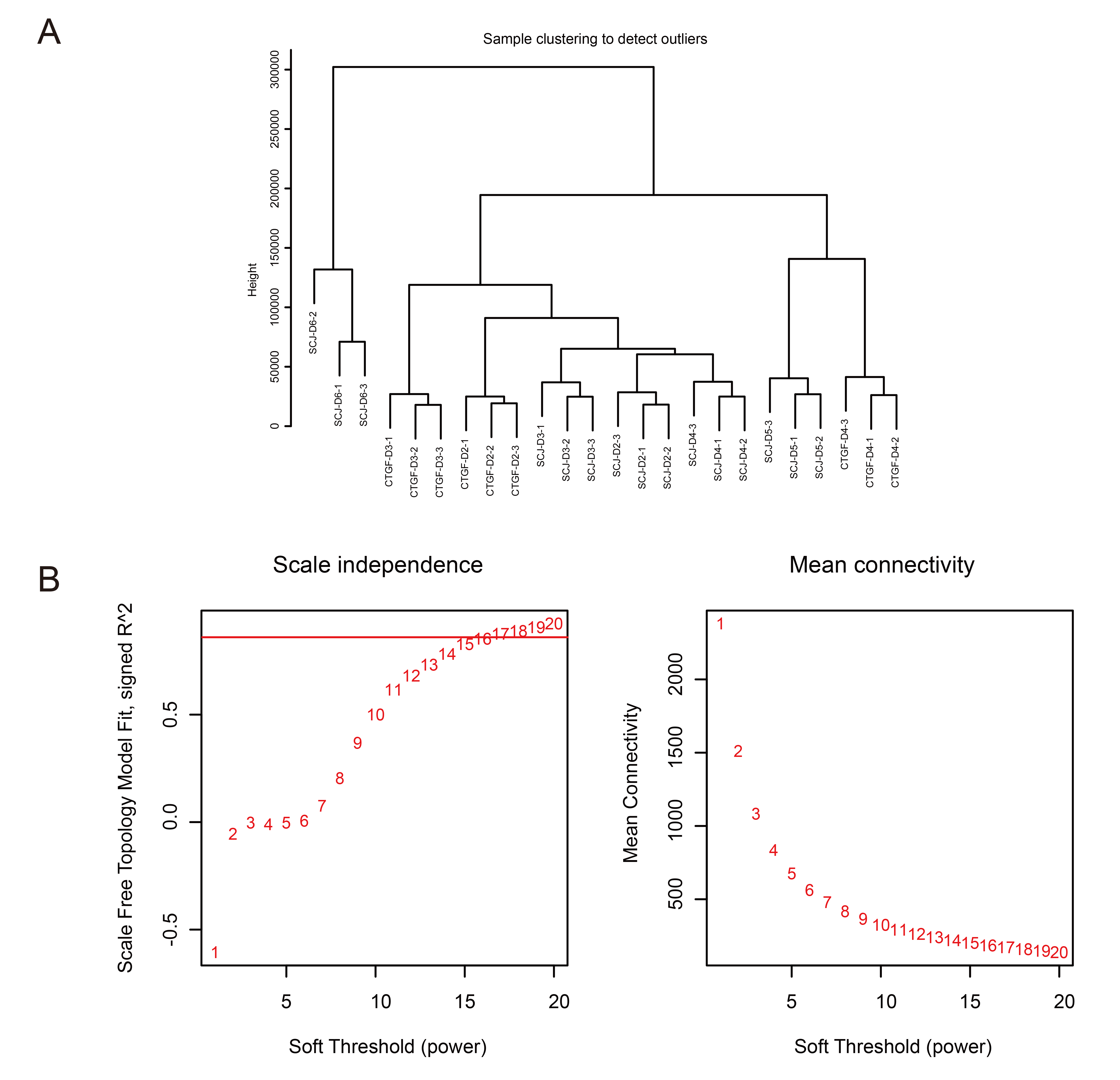

Supplement: Supplementary file 1 [file plants-15-01379-s001.zip › Figure S4.tif]

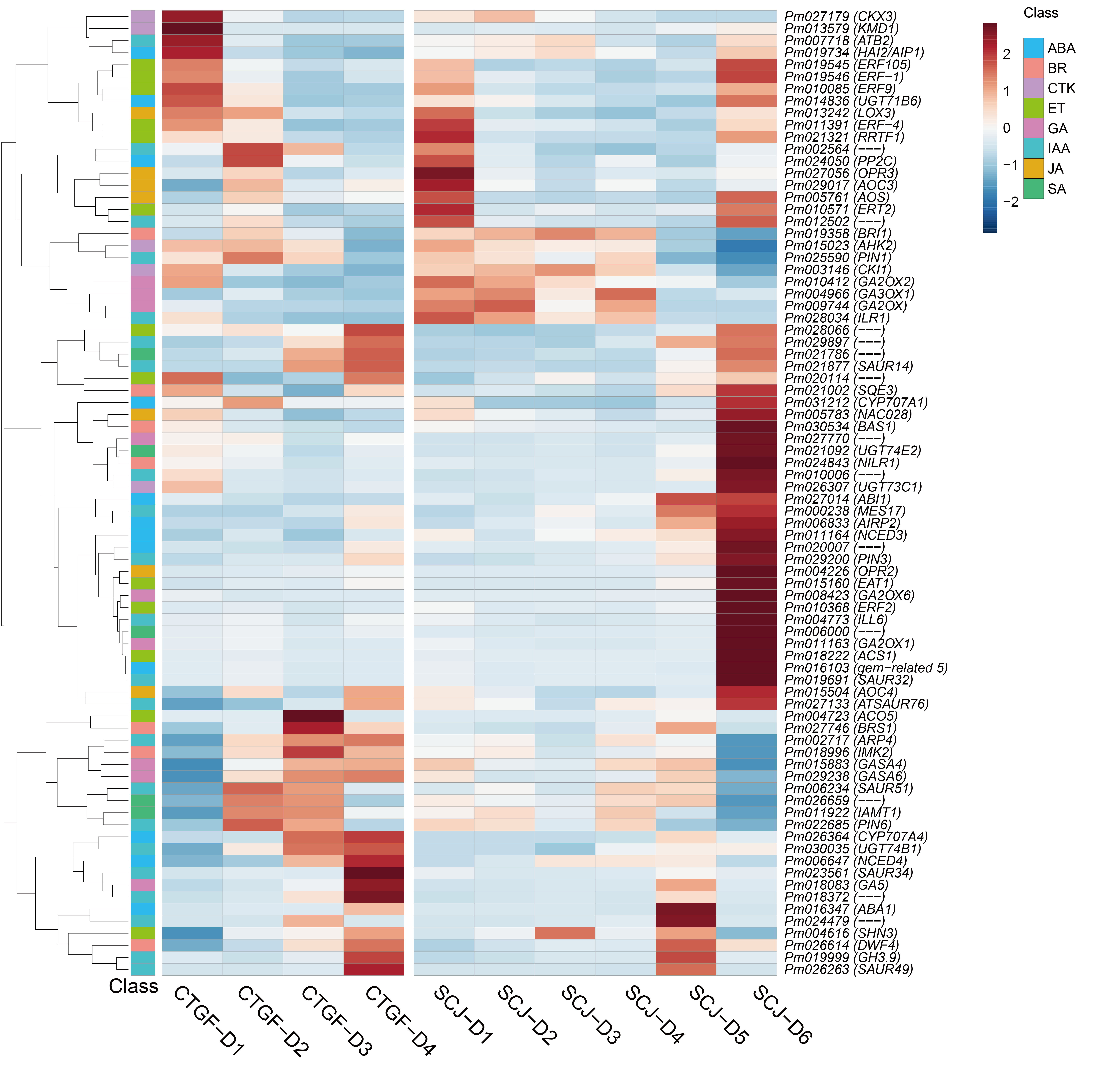

Supplement: Supplementary file 1 [file plants-15-01379-s001.zip › Figure S5.tif]

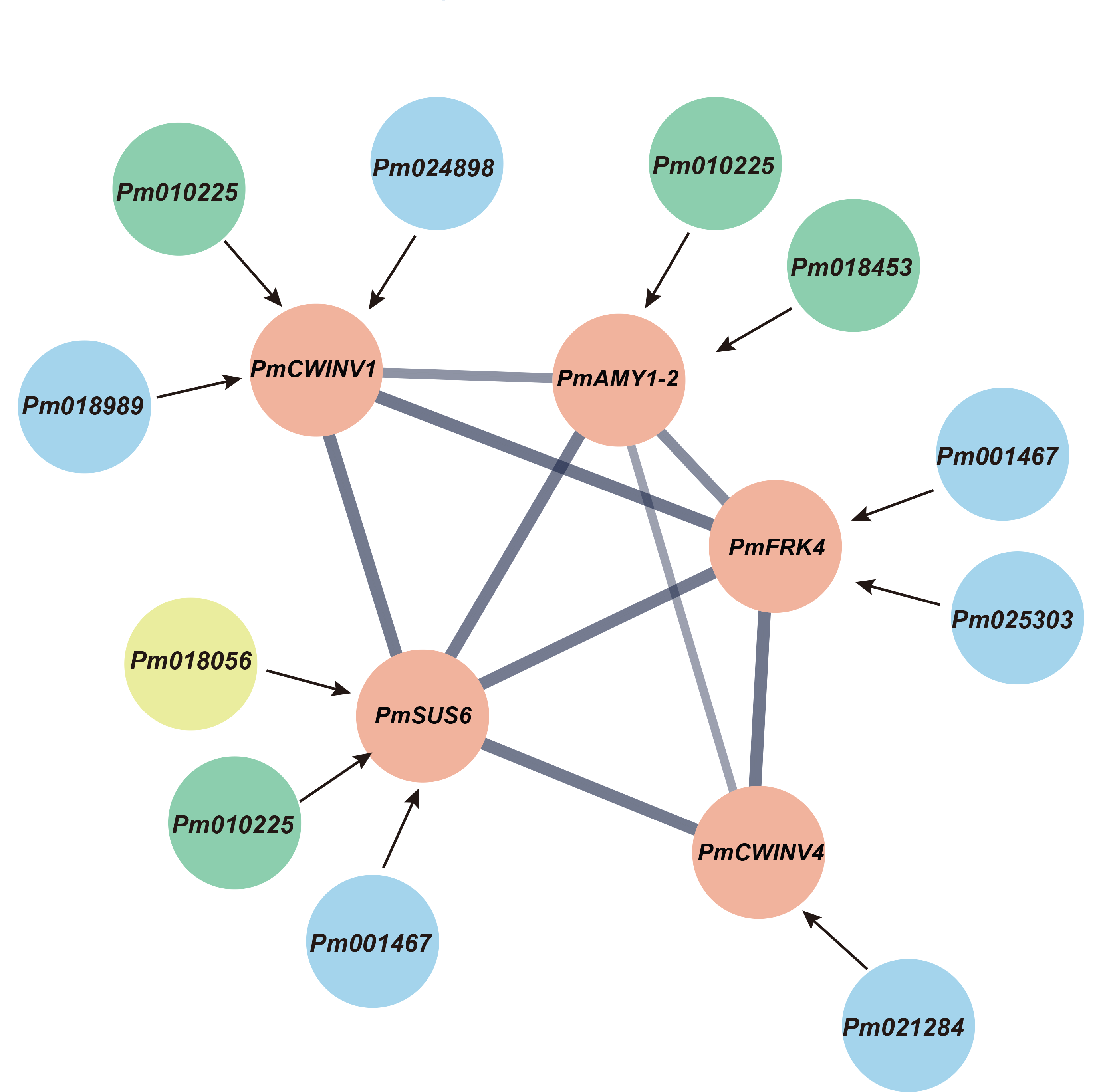

Supplement: Supplementary file 1 [file plants-15-01379-s001.zip › Figure S6.tif]
